# Supplementary material for: Causal Relationship between Aging and Anorexia Nervosa: A White-Matter-Microstructure-Mediated Mendelian Randomization Analysis
Source: Biomedicines. 2024 Aug 16;12(8):1874. doi: 10.3390/biomedicines12081874 (PMC11351342; doi:10.3390/biomedicines12081874)
Supplement: Supplementary file 1 [file biomedicines-12-01874-s001.zip › Supplementary Figure/Figure S4/Singe SNP plot of MR analysis for TL to ubm-b-1694.pdf]

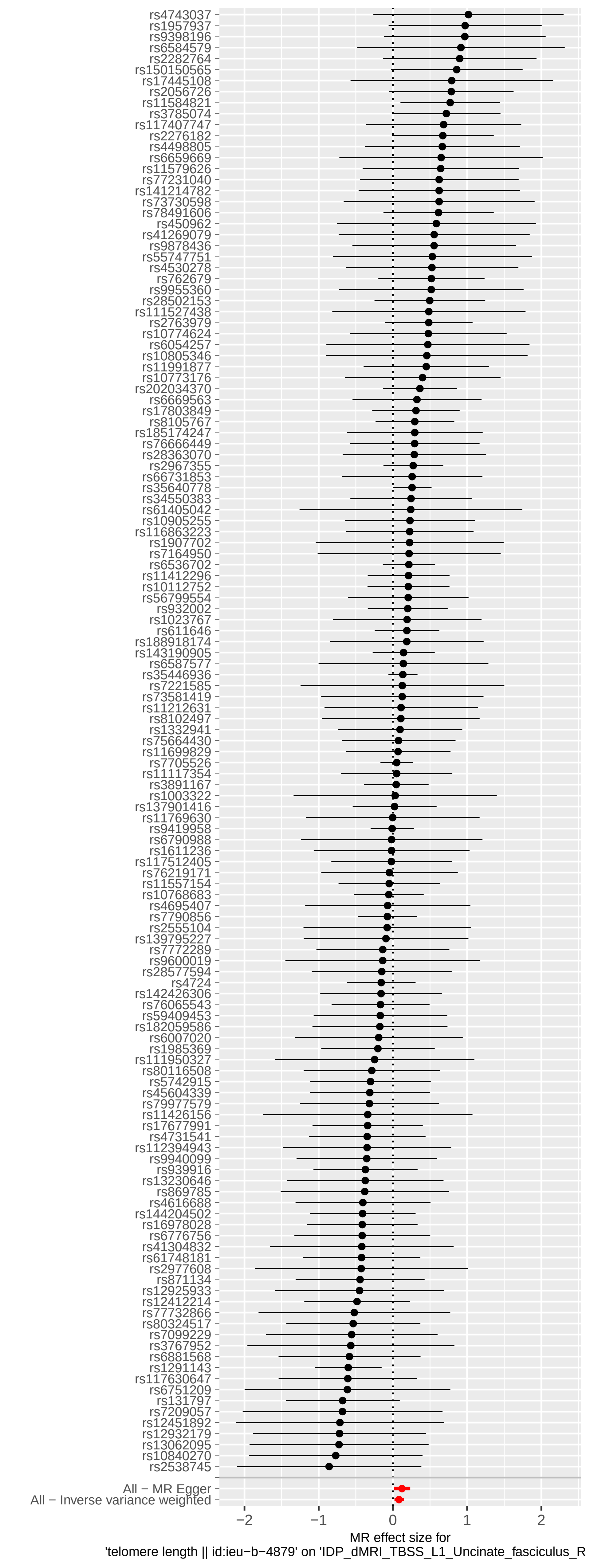
rs4743037
rs1957937
rs9398196
rs6584579
rs2282764
rs150150565
rs17445108
rs2056726
rs11584821
rs3785074
rs117407747
rs2276182
rs4498805
rs6659669
rs11579626
rs77231040
rs141214782
rs73730598
rs78491606
rs450962
rs41269079
rs9878436
rs55747751
rs4530278
rs762679
rs9955360
rs28502153
rs111527438
rs2763979
rs10774624
rs6054257
rs10805346
rs11991877
rs10773176
rs202034370
rs6669563
rs17803849
rs8105767
rs185174247
rs76666449
rs28363070
rs2967355
rs66731853
rs35640778
rs34550383
rs61405042
rs10905255
rs116863223
rs1907702
rs7164950
rs6536702
rs11412296
rs10112752
rs56799554
rs932002
rs1023767
rs611646
rs188918174
rs143190905
rs6587577
rs35446936
rs7221585
rs73581419
rs11212631
rs8102497
rs1332941
rs75664430
rs11699829
rs7705526
rs11117354
rs3891167
rs1003322
rs137901416
rs11769630
rs9419958
rs6790988
rs1611236
rs117512405
rs76219171
rs11557154
rs10768683
rs4695407
rs7790856
rs2555104
rs139795227
rs7772289
rs9600019
rs28577594
rs4724
rs142426306
rs76065543
rs59409453
rs182059586
rs6007020
rs1985369
rs111950327
rs80116508
rs5742915
rs45604339
rs79977579
rs11426156
rs17677991
rs4731541
rs112394943
rs9940099
rs939916
rs13230646
rs869785
rs4616688
rs144204502
rs16978028
rs6776756
rs41304832
rs61748181
rs2977608
rs871134
rs12925933
rs12412214
rs77732866
rs80324517
rs7099229
rs3767952
rs6881568
rs1291143
rs117630647
rs6751209
rs131797
rs7209057
rs12451892
rs12932179
rs13062095
rs10840270
rs2538745
All − MR Egger
All − Inverse variance weighted
−2
−1
0
1
2
MR effect size for
'telomere length || id:ieu−b−4879' on 'IDP_dMRI_TBSS_L1_Uncinate_fasciculus_R
